# Effective Extraction and Assembly Methods for Simultaneously Obtaining Plastid and Mitochondrial Genomes

**DOI:** 10.1371/journal.pone.0108291

**Published:** 2014-09-24

**Authors:** Wanjun Hao, Shihang Fan, Wei Hua, Hanzhong Wang

**Affiliations:** 1 Key Laboratory for Biological Sciences of Oil Crops, Ministry of Agriculture, Oil Crops Research Institute, Chinese Academy of Agriculture Sciences, Wuhan, China; 2 College of Life Sciences, Hubei University, Wuhan, China; University of California - Davis, United States of America

## Abstract

**Background:**

In conventional approaches to plastid and mitochondrial genome sequencing, the sequencing steps are performed separately; thus, plastid DNA (ptDNA) and mitochondrial DNA (mtDNA) should be prepared independently. However, it is difficult to extract pure ptDNA and mtDNA from plant tissue. Following the development of high-throughput sequencing technology, many researchers have attempted to obtain plastid genomes or mitochondrial genomes using high-throughput sequencing data from total DNA. Unfortunately, the huge datasets generated consume massive computing and storage resources and cost a great deal, and even more importantly, excessive pollution reads affect the accuracy of the assembly. Therefore, it is necessary to develop an effective method that can generate base sequences from plant tissue and that is suitable for all plant species. Here, we describe a highly effective, low-cost method for obtaining plastid and mitochondrial genomes simultaneously.

**Results:**

First, we obtained high-quality DNA employing Partial Concentration Extraction. Second, we evaluated the purity of the DNA sample and determined the sequencing dataset size employing Vector Control Quantitative Analysis. Third, paired-end reads were obtained using a high-throughput sequencing platform. Fourth, we obtained scaffolds employing Two-step Assembly. Finally, we filled in gaps using specific methods and obtained complete plastid and mitochondrial genomes. To ensure the accuracy of plastid and mitochondrial genomes, we validated the assembly using PCR and Sanger sequencing. Using this method,we obtained complete plastid and mitochondrial genomes with lengths of 153,533 nt and 223,412 nt separately.

**Conclusion:**

A simple method for extracting, evaluating, sequencing and assembling plastid and mitochondrial genomes was developed. This method has many advantages: it is timesaving, inexpensive and reproducible and produces high-quality sequence. Furthermore, this method can produce plastid and mitochondrial genomes simultaneously and be used for other plant species. Due to its simplicity and extensive applicability, this method will support research on plant cytoplasmic genomes.

## Introduction

The majority of plant progenies inherit their plastid and mitochondrial DNA from the maternal parent, and in recent decades, plastid and mitochondrial genomes have been used widely in studies on diversity and evolution. Moreover, plastids and mitochondria are important energy and metabolism organelles in plant cells. Many anabolic and catabolic processes occur in these two organelles, such as photosynthesis, respiration, and fatty acid synthesis. Thus, plastid and mitochondrial DNA have recent particular attention in plant research, highlighting the need to obtain plastid and mitochondrial genomic sequences.

Conventional approaches to generating plastid and mitochondrial genome sequences use separate processes. Thus, plastid DNA (ptDNA) and mitochondrial DNA (mtDNA) are prepared independently. Typically, researchers purify ptDNA and mtDNA from green leaves and etiolated seedlings separately employing density-gradient ultracentrifugation (CsCl, sucrose, or percol) [1]–[4]. This demanding protocol is unsuitable for wide use for plant plastid and mitochondrial genome sequencing. An additional method uses Long-PCR to amplify ptDNA or mtDNA prior to sequencing. In recent years, high-throughput sequencing platforms have been used to capture sequence data from many individual PCR amplifications that cover the entire plastid or mitochondrial genome [5], [6]. Because this method requires a reference sequence, it can be used only for a few species; moreover, it is time consuming. Thus, many researchers attempted to obtain plastid genomes or mitochondrial genomes using high-throughput sequencing data from total DNA. Unfortunately, the huge datasets generated consume massive computing and storage resources and cost a great deal, and even more importantly, excessive false-positive reads affect the accuracy of the assembly.

Here, we report a simple method that extracts, evaluates, sequences and assembles plastid and mitochondrial genomes simultaneously. Using this method, first, crude plastids and mitochondria were isolated together employing differential centrifugation. Subsequently, ptDNA and mtDNA were extracted from this crude preparation of plastids and mitochondria. Following their evaluation, eligible DNA samples were used for high-throughput sequencing. Finally, the complete plastid and mitochondria genome sequences were obtained employing de novo assembly. This method is economical and timesaving and can be used for all species.

## Materials and Methods

### Plant material

*Brassica napus* L. line DH366, a fertile rapeseed line possessing Polima cytoplasm, was used for this study. Rapeseed seeds were surface sterilized using 70% ethanol for 2 min, treated with 10% sodium hypochlorite for 15 min, and subsequently washed 4–5 times using sterile water. The sterile seeds were inoculated in 150 ml Erlenmeyer flasks containing 1/2 MS media and incubated in the dark at 22°C and 70% relative humidity. Four-week-old etiolated seedlings were collected for DNA extraction.

### DNA extraction

#### Reagents and solutions

Homogenization medium: 0.4 M mannitol, 1 mM EDTA, 25 mM MOPS-KOH, 10 mM tricine, 8 mM cysteine, 0.1% BSA and 0.1% PVP-40, pH 7.8.

Extraction medium: 50 mM Tris-HCl, 10 mM EDTA, 2% sarkosyl and 0.012% Proteinase K, pH 8.0.

TE: 10 mM Tris-HCl and 1 mM EDTA, pH 8.0.

#### Protocols

Step one to five were conducted at 0°C, and all equipment, consumables and reagents were cooled to 0°C.

1. Etiolated seedlings (5 g, fresh weight) were chopped up using a pair of scissors in 50 ml of pre-cooled homogenization medium.
2. The chopped tissue was then transferred into a Dounce tissue grinder and ground 80 times in an ice bath.
3. The homogenate was poured into a 50 ml centrifuge tube and centrifuged for 10 min at 500 g to remove nucleus and cell fragments.
4. The supernatant was transferred into a new 50 ml centrifuge tube and centrifuged for 5 min at 1000 g to remove residual contamination.
5. The supernatant was transferred into a new 50 ml centrifuge tube and centrifuged for 20 min at 12,000 g to precipitate the plastids and mitochondria.
6. The supernatant was discarded, and the pellet was composed of crude plastids and mitochondria.
7. Extraction medium (1.5 ml) was added to the crude pellets and mixed using pipette tips, and the mixture was then transferred into two 2 ml centrifuge tubes.
8. The tubes were capped and the suspensions incubated at 37°C for 1 hour to disrupt the organelles.
9. The tubes were cooled at room temperature for 5 min, and 85 µl of 2 M sodium acetate solution was then added to each tube.
10. Equilibrated phenol (850 µl) was added to each tube, and the solutions mixed well and then centrifuged at 13,200 rpm for 10 min.
11. The upper aqueous phase was transferred into new 1.5 ml centrifuge tubes, an equivalent volume of chloroform:isoamylalcohol (24∶1, v/v) was added to each tube, and the solutions were mixed well and then centrifuged at 13,200 rpm for 10 min.
12. The upper aqueous phase was transferred to new 1.5 ml centrifuge tubes, 2-fold volume of 100% ethanol was added, and the contents were mixed well and then incubated at −20°C for 30 min to dissociate the DNA.
13. The tube was centrifuged for 5 min at 13,200 rpm, and the supernatant was removed.
14. The precipitated DNA was washed twice with 70% ethanol.
15. The DNA was dried at room temperature and re-dissolved in 50 µl TE buffer.

### DNA evaluation

**Figure 1:**
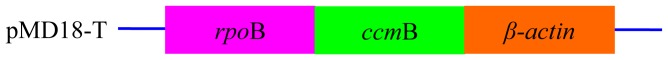
The control pMD18-T-VCQA vector.



In this study, a new method, Vector Control Quantitative Analysis (VCQA), was used for DNA evaluation. First, three genes–*rpoB, ccmB* and *β-actin*–were cloned from the rapeseed plastid, mitochondrial and nuclear genomes separately. Second, three genes were joined into one fragment using overlap extension PCR. Third, the synthetic fragment was cloned into the pMD-18 vector to obtain the control vector pMD18-T-VCQA (Figure 1). Fourth, the copy folds of *rpoB/β-actin* and *ccm*B/*β-actin* were determined employing qPCR. Finally, we used *rpoB, ccmB* and *β-actin* to represent ptDNA, mtDNA and ncDNA separately. Thus, the copy fold of organelle DNA to ncDNA in each DNA sample could be computed using the following equation:
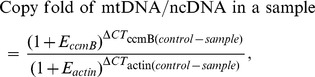



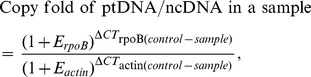
where E = efficiency of amplification and CT = number of cycles. The detailed derivations of these equations are described in File S1.

### DNA sequencing

Approximately 3 µg of purified DNA was used to prepare sequence libraries. A 500 bp DNA library was prepared following the manufacturer’s instructions (Illumina sample preparation protocol for paired-end sequencing). This DNA library was sequenced over 100×2 cycles on an Illumina HiSeq2000 sequencing platform (http://www.illumina.com). Base calling was performed using Illumina Pipeline 1.8 (Illumina, San Diego, CA, US). We then discarded read pairs in which the reads contained adaptor sequences, more than 10% unknown bases (N) or more than 50% low-quality (<5) bases. All of this work was performed by Novagene (Beijing, China).

### *De novo* assembly

In this study, assembly was conducted using the Two-step Assembly (TSA) method. A detailed description of TSA follows.

**Figure 2:**
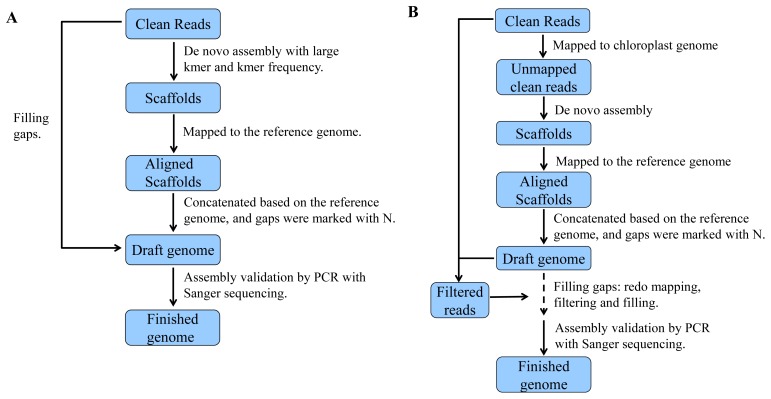
Flowchart showing the major steps of de novo assembly employing TSA. A) First step: assembly of the plastid genome; B) second step: assembly of the mitochondrial genome.

During the first step, the plastid genome was assembled. The detailed pipeline is shown below (Figure 2A). 1) De novo assembly: de novo assembly was performed using SOAPdenovo2 software [7] using high kmer size and kmer frequency to eliminate the effects of plastid reads and nuclear reads. 2) Scaffold mapping: we mapped all the scaffolds to the reference plastid genome using BLAT software [8]. The mapped scaffolds were filtered and used to construct the draft genome. 4) Constructing the draft genome: all mapped scaffolds were ordered manually based on their position in the reference genome and were connected into a draft genome using overlapping information with gaps filled using N. 5) Gap closure: we filled gaps with all the reads employing the GapCloser software in SOAPdenovo2. 6) Assembly validation: we validated the order of the scaffolds and accuracy of the sequence employing PCR amplification and Sanger sequencing.

For the second step, we conducted de novo assembly of the mitochondrial genome. The detailed pipeline is shown below (Figure 2B). 1) Filtering plastid reads: we mapped the total clean reads to the plastid genome obtained above using SOAP2 software (http://soap.genomics.org.cn/soapaligner.html) with no mismatch. Only unmapped pair-end reads were used for the next step. 2) De novo assembly: the de novo assembly was performed using SOAPdenovo2 software. 3) Scaffold mapping: we mapped all the scaffolds to the reference mitochondrial genome using BLAT software. The mapped scaffolds were filtered and used to construct the draft genome. 4) Constructing the draft genome: all the mapped scaffolds were ordered manually and connected into a draft genome based on their position in the reference genome using overlap information with gaps filled with N. 5) Gap closure: because plastid reads were pre-removed prior to mitochondrial assembly, theoretically, any region similar to the plastid genome in a mitochondrial genome could create a gap. To ensure accuracy, we filled gaps employing a strict method. First, all reads were mapped to the draft genome. Then, paired-end reads, of which at least one end mapped to the draft genome exactly, were filtered. Finally, filtered reads were used to fill gaps employing GapCloser software. Read mapping, read filtering and gap filling continued until all gaps no longer extended. The remaining gaps were filled in using PCR amplification and Sanger sequencing. 6) Assembly validation: we validated the order of the scaffolds and accuracy of the sequence employing PCR amplification and Sanger sequencing.

### Gene annotation and variation analyses

When we obtained the complete plastid genome and mitochondrial genome, genes were annotated using DOGMA (http://dogma.ccbb.utexas.edu/) [9]. Using their respective reference genomes, plastid and mitochondrial genomes were compared for variations using Crossmatch in Phrap (http://www.phrap.org/). All the variations were then annotated using snpEff software [10].

## Results and Discussion

### DNA extraction using PCE

The conventional approach used to prepare ptDNA or mtDNA samples for sequencing is quite complex. The method requires density gradient ultracentrifugation and large amounts of green leaves or etiolated seedlings [1]–[4]. In recent years, alternative methods have been developed, such as differential centrifugation combining DNase I digestion [1], [4], [11]–[19], Long-PCR [5], [6], multiply-primed rolling circle amplification (RCA) [20] and a probe enrichment strategy [21]. All of these methods have deficiencies, such as their complexity, instability or lack of suitability for wide use. Most importantly, no method can extract ptDNA and mtDNA together except when total DNA is used. In this study, we developed a method that can enrich ptDNA and mtDNA simultaneously. Because the method can only increase the proportion of ptDNA and mtDNA in a DNA sample, we named it Partial Concentration Extraction (PCE).

**Figure 3:**
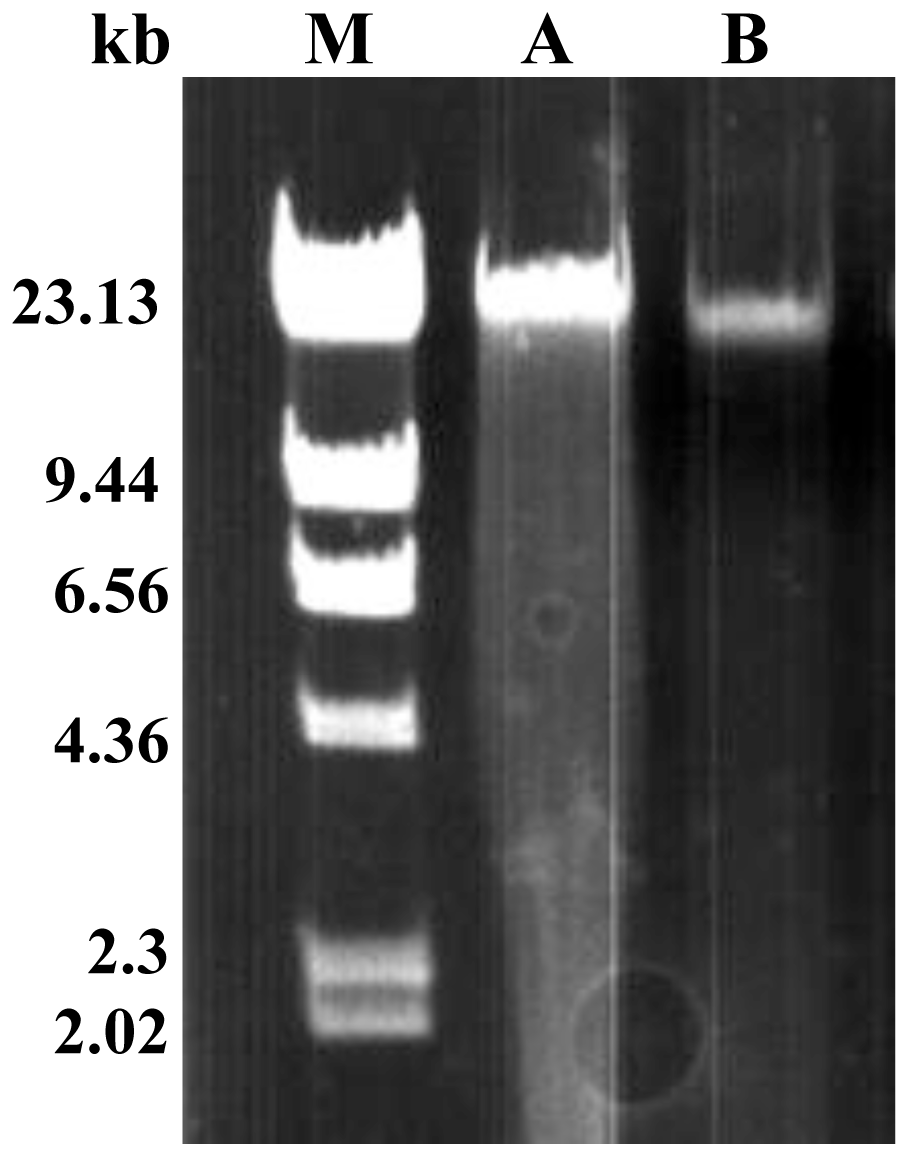
Comparison of DNA extracted with CTAB versus PCE. A) Total DNA extracted with CTAB and B) extracted with PCE; M denotes Lambda DNA/Hind III.

Using PCE, first, crude plastids and mitochondria were isolated employing differential centrifugation from etiolated seedlings. Then, ptDNA and mtDNA were extracted using phenol-chloroform extraction and ethanol precipitation. The ptDNA and mtDNA were enriched in the resulting DNA. The details of the DNA extraction are described in the Materials and Methods. Finally, a total of 7.17 µg DNA was obtained from 5 g of etiolated seedlings. The results in Table 1 show the DNA quality examined using a NanoDrop 1000. The quality of the DNA was further tested using electrophoresis of the DNA on a 1% agarose gel. As shown in Figure 3, no degradation of the DNA fragment extracted using PCE was observed. All the results indicate that we obtained a high-quality DNA sample suitable for sequencing.

**Table 1:** Evaluation of DNA quality using a NanoDrop 1000.

Sample ID	ng/µl	A260	A280	260/280	260/230
DH366	143.31	2.866	1.479	1.94	2.22

During the entire procedure, no density gradient ultracentrifugation steps, DNase I digestion, PCR, special probes or reagents were used. Compared with the relatively simple method using differential centrifugation [17], this method saves time and requires less tissue (Table 2). However, to obtain sufficient and intact plastids and mitochondria, an essential precondition is the use of a Dounce tissue grinder and suitable homogenization medium. The etiolated seedlings can decrease the friction drag and facilitate tissue grinding. The simplicity of this method provides it with a higher success rate than others methods. Moreover, this method can be widely used in most laboratories and all plant species. More importantly, when combined with our assembly method, this method easily obtains plastid and mitochondrial genomes simultaneously at little cost.

**Table 2:** Comparison between partial concentration extraction and differential centrifugation.

Method	Partial concentration extraction	Differential centrifugation [17]	
		ptDNA	mtDNA
Tissue Type	etiolated seedlings	leaves	
Total Tissue	5 g	5 g	10–15 g
Total Time	∼4 h	>4 h	∼2.5 h

### Purity evaluation using VCQA

Many studies indicate that sequence homology exists between the plastid, mitochondrial and nuclear genome. Thus, when de novo assembly is performed, the plastid genome should influence the mitochondrial genome and vice versa, and both the plastid and mitochondrial genomes should be affected by the nuclear genome. In the past, many studies used PCR or restriction digestion to perform qualitative analysis of ncDNA in pure ptDNA and mtDNA preparations; however, no method can perform quantitative analysis [12], [18], [19]. VCQA, a method based on qPCR, can perform quantitative analysis of DNA types in a DNA sample. For this method, we used a control vector, in which three genes–*rpoB, ccmB* and *β-actin*–belong to plastid, mitochondrial and nuclear genomes, respectively, and represent ptDNA, mtDNA and ncDNA separately. Thus, the copy folds of ptDNA/ncDNA and mtDNA/ncDNA in the DNA sample can be easily identified employing quantitative analysis of the three genes.

We evaluated the purity of the DNA sample using VCQA and predicted the sequencing data size for a given sequencing depth. The detailed results are listed in Table 3. In our DNA sample, the copy fold of mtDNA/ncDNA is 72, whereas the copy fold of ptDNA/ncDNA is, astonishingly, 1922. Thus, when the sequencing data cover the entire genome (approximately 1.2 GB) one layer, the mitochondrial genome is 72 layers and the plastid genome is 1922 layers. In theory, an ideal de novo assembly can be obtained when the sequencing depth is greater than 50 layers. Thus, if the sequencing dataset size reaches 1.2 GB, it is sufficient for de novo assembly of mitochondrial and plastid genomes in our DNA sample. These data suggest that our method produces high-purity DNA samples for sequencing plastid mitochondrial genomes.

**Table 3:** Quantitative analysis of DNA purity using VCQA employing qPCR.

Genes	E	pMD18-T-VCQA	DH366	ΔCT_(control-sample)_	(1+E)^ΔCT(control-sample)^	*ccmB/actin*	*rpoB/actin*
*actin*	1.124	17.79	23.70	−5.91	0.0117	–	–
*ccmB*	1.060	17.64	17.87	−0.23	0.8469	72	–
*rpoB*	1.042	17.76	13.40	4.36	22.4824	–	1922

VCQA = Vector Control Quantitative Analysis; E = efficiency of amplification; CT = number of cycles.

Because the method can be used for quantitative analysis, it can be used to compare the DNA of plastids and mitochondria from different materials when performing correlation analyses of ptDNA/mtDNA content and phenotypes.

### Genome sequencing and sequencing depth statistics

In this study, a whole-genome shotgun strategy and HiSeq2000 sequencing platform were employed. Paired-end sequencing libraries with insert sizes of approximately 500 bp were constructed using 3 µg DNA samples. Sequencing reads containing adaptor sequences were cleaned, and the sequence data were filtered for low-quality reads. This process resulted in a total of 5,778,987 high-quality paired-end reads containing 1,155,797,400 bases with an average read size of 100 nt.

To obtain satisfactory assemblies, we computed the accurate sequencing depths of the mitochondrial and plastid genomes using the mitochondrial genome (Accession number FR715249) of 2H2A and the plastid genome (Accession number GQ861354) of ZY036 as references. First, we mapped the high-quality reads to the reference genome. Then we computed the sequencing depths of target genes (*rpoB* and *ccmB*) and unique regions excluding similar regions of plastid and mitochondrial genomes. The detailed results are listed in Table 4.

**Table 4:** Summary of sequence alignments and sequencing depths.

Items	Plastid	*rpoB*	Mitochondrial	*ccmB*
Reference length^a^	142,995	3,219	216,086	621
Mapped reads	2,786,560	6,9475	199,077	520
Mapped bases	278,656,000	6,947,500	19,907,700	52,000
Sequencing depth	1,949	2,158	92	84

a Reference: plastid (Accession number GQ861354), mitochondrial (Accession number FR715249); only unique regions were used.

The sequencing depths of unique regions of the plastid and mitochondrial genomes are similar to the sequencing depths obtained for *rpoB* and *ccmB* separately, with differences of less than 10% observed. The sequencing data were nearly 1.2 GB and covered the nuclear genome to approximately one layer. Thus, the sequencing depth of the plastid and mitochondrial genomes can be expected to be equivalent to the copy folds of ptDNA/ncDNA and mtDNA/ncDNA separately. Indeed, the sequencing depths of unique regions and target genes (*rpoB* and *ccmB*) are consistent with the copy folds, suggesting that this method of DNA evaluation is highly reliable.

We compared the sequencing depth of our DNA sequencing data and total DNA sequencing data. One sample was part of the whole genome sequencing data of total rapeseed DNA (unpublished). The additional two samples were from previous studies [22], [23]. When the nuclear genome was covered one layer, the sequencing depth of the plastid genome in our sample was 15–35-fold that of the three total DNA samples, and the sequencing depth of the mitochondrial genome in our sample was approximately 4–9-fold that of the three total DNA samples (Table 5). These data suggest that our DNA extraction method yielded a highly pure DNA sample for sequencing the plastid and mitochondrial genomes.

**Table 5:** Comparison of sequencing depth in different DNA samples.

Sample	ptDNA/ncDNA^a^	mtDNA/ncDNA^b^	Literature cited
Our DNA	1,949	92	This report
Total DNA 1^c^	117	23	unpublished
Total DNA 2	55	11	[22]
Total DNA 3	134	11	[23]

a Copy fold of plastid DNA (ptDNA) to nuclear DNA (ncDNA);

b Copy fold of plastid DNA (ptDNA) to nuclear DNA (ncDNA);

c Total DNA of *Brassica napus* L.

### *De novo* assembly using TSA

With the use of the high-throughput sequencing platform, many researchers have attempted to use total DNA to obtain plastid genome or mitochondrial genome sequences; however, the assembly accuracy has never achieved a satisfactory resolution. In some cases, plastid reads were initially separated from total reads employing the published plastid genomes [24], [25] and then used for de novo assembly of plastid genomes. However, the sequence differences between different materials, in particular, the large insertions and deletions, affect the efficiency of the assembly and lead to more gaps. When assembling mitochondrial genomes, plastid reads were removed from total reads employing the existing plastid genomes [23]. Sequence differences in plastid genomes between different materials prevent the removal of partial plastid reads and lead to false assembly. In some studies [26], the contigs were filtered employing the different sequencing depths of plastids, mitochondria and nucleus. However, this approach cannot avoid false de novo assembly of contigs.

To avoid false assembly, we developed a new assembly method, TSA, that is used with the PCE DNA extraction method in combination. In our sequencing data, the sequencing depth of the nuclear genome is approximately one layer, which is far lower than the sequencing depths of plastid and mitochondrial genomes. Thus, the effect of nuclear reads can be ignored when performing de novo assembly of plastid and mitochondrial genomes. Despite the sequencing depth of the mitochondrial genome reaching almost 100 layers, it remains very low when compared with the 2000 layers obtained for the plastid genome. Thus, we can easily eliminate the effects of mitochondrial reads by employing the large kmer and kmer frequency when performing de novo assembly of the plastid genome.

Based on the depth evaluated above, de novo assembly was performed using a series of different parameters. Finally, we obtained an optimal result. Only six scaffolds (>1 kb) were assembled, and three scaffolds were aligned to the reference plastid genome (Accession number GQ861354; Table 6). Three aligned scaffolds were then ordered manually and connected into a draft sequence based on positions in the reference genome using overlap information with gaps filled with N. To obtain the finished sequence, all the reads was used to fill gaps employing GapCloser. Finally, we obtained a complete plastid genome with a length of 153,533 nt (Table 6) including a pair of inverted repeats (IRs) of 26,186 nt separated by one small and one large single-copy region (SSC and LSC) of 17,780 and 83,381 nt, respectively.

**Table 6:** Summary of de novo assembly results.

Genome	Scaffold number (Total/Aligned)^a^		Gap number		Total length (nt)
	>1 kb	>10 kb	Total	Filled	
Plastid	6/3	3/3	1	1	153,533
Mitochondrial	44/4	3/3	2	2	223,412

*Note:* Reference plastid genome (Accession number GQ861354), reference mitochondrial genome (Accession number FR715249).

The mitochondrial genome assembly was more complicated than that of the plastid genome. The sequencing depth of the plastid genome was far greater than for the mitochondrial genome, and we cannot eliminate effects of plastid reads employing sequencing depth. To reduce the effects of the plastid reads, we must remove the plastid reads from the total reads. We used the plastid genome obtained above as a reference to avoid sequence differences between the different materials. When at least one end mapped exactly to the plastid genome, we considered the paired-end read as a plastid read and removed it. Finally, 4,165,859 paired-end reads, accounting for 72% of the total reads, remained and were used for the de novo assembly of the mitochondrial genome.

After several attempts, we assembled the filtered reads using the following optimal parameters: kmer size 41, kmer frequency 20 and edge coverage 20. Finally, 44 scaffolds greater than 1 kb were obtained and mapped to the reference mitochondrial genome (Accession number FR715249), resulting in four mapped scaffolds (Table 6). The mapped scaffolds were ordered manually and connected into a draft genome based on their positions in the reference genome using overlap information with gaps filled with N. To ensure the accuracy of the sequence, we filled gaps employing a rigorous method. In the first cycle, we mapped the total reads to the draft genome, and 496,252 paired-end reads with at least one end were mapped and filtered. The filtered reads were used to fill gaps, and one gap was filled. During the second cycle, we mapped the total reads to the sequence obtained after the first cycle. Consequently, 594,260 paired-end reads were mapped and were used to fill the remaining gaps. After two cycles of read mapping, read filtering and gap filling, all gaps were filled. Ultimately, we obtained the complete mitochondrial genome sequence of 223,412 nt in length (Table 6).

We used PCR and Sanger sequencing to confirm the sequence accuracy of this novel method, which has been used for the first time to assemble plastid and mitochondrial genomes. We designed ten pairs of primers to amplify the sequence fragment, which contained several junction regions of two scaffolds (Table 7). Following PCR, we identified the sequence of the twelve PCR products using Sanger sequencing. The sequences obtained were compared with the complete genome. No false results were obtained in ten regions adding up to 8,071 bp (Table 7). These data indicate the high quality of the de novo assembly and the high accuracy of the order of scaffolds and gap filling.

**Table 7:** Primer pairs used for assembly validation by PCR using Sanger sequencing.

Forward	Sequence(5′->3′)	Reverse	sequence(5′->3′)	Product (bp)	Identity (%)
CP1-F	TCCGTGCTTTGTGGGCAGAC	CP1-R	GCAGCGGGTGCTGTAGCGAA	843	100
CP2-F	AGGAACCTCAGAAACGGGTGGGA	CP2-R	ACGCCGTCGATAAACCTTTTGCAT	730	100
MT1-F	TCTACGTTTCACACGACGCA	MT1-R	GAGATGCGGGTAGAGGAAGC	897	100
MT2-F	AACCCCGGAGCTCTTCAATG	MT2-R	GTTTTGGGTACAGAGGGCCA	1306	100
MT3-F	CACTAACTCTGCCTGGGGTG	MT3-R	GCAGTTGGAAGTTGCTTGGG	698	100
MT4-F	TAAAGGCTGGGCGAAGGGAG	MT4-R	CACACGAAAGGGAACGAGGA	533	100
MT5-F	AAGAGGCCGATAGGACCAGT	MT5-R	CCTCTTGGCAGCCTTCTTCA	824	100
MT6-F	GTGCGAAAGGGCTTGTTTGT	MT6-R	GACCCCACCGTAGAAAGAGC	652	100
MT7-F	CTAGCGCTTCGCGTTCTTTC	MT7-R	TGGCTGTTGGTCACGACTAC	849	100
MT8-F	GTTCTGTTCCTGCCACGAGA	MT8-R	GGCAGAGCACGAGGAGATTT	739	100

### Gene annotation and comparative analysis

The first plastid genome of the Polima cytoplasm was annotated using DOGMA. The detailed annotation information was submitted to GenBank (KJ872515). This genome contains the same gene number and order as the ZY036 plastid genome, which possesses a nap cytoplasm. However, when compared with ZY036, the plastid genome is 81 nt longer and there are 202 single-nucleotide polymorphisms (SNPs), 5 multi-nucleotide polymorphisms (MNPs), 106 insertions and deletions (indels) and 13 complex variations (CVs). We annotated all of these variations based on gene information from ZY036. Seventy-one SNPs were located in the coding region of 23 genes, and 31 SNPs changed the amino acid sequences of 10 genes. The indels ranged from 1 nt to 108 nt, with an average length of 5 nt. No indel, MNP or CV was located in coding regions (Table 8).

**Table 8:** Variations in plastid and mitochondrial genomes.

Organelles	Types	Number	Coding region	Non-synonymous
Plastid	SNP	202	71	31
	Indel	106	0	0
	MNP^a^	5	0	0
	CV^b^	13	0	0
Mitochondrial	SNP	4	2	1

*Note:* Reference plastid genome (Accession number GQ861354), reference mitochondrial genome (Accession number FR715249).

a MNP: multiple nucleotide polymorphisms;

b CV: complex variations.

For the first time, we report on the mitochondrial genome of a fertile rapeseed line that possesses a Polima cytoplasm. We compared it with the mitochondrial genome of a Polima cytoplasmic male-sterile line Shaan 2A [14]. Surprisingly, the mitochondrial genome of DH366 exhibited the same length as Shaan 2A. Only four SNPs were noted, of which only two were located in coding regions of two genes and only one SNP changed the amino acid sequence of ORF257 (Table 8). With respect to the exiguous variations, the mitochondrial genome of DH366 possesses the same gene number as Shaan 2A, including 34 protein encoding genes, three ribosomal RNA genes, 18 transfer RNA genes and 40 putative open reading frames (ORFs).

Similar to the plastid genome, enormous variations were observed in the mitochondrial genome when the nap cytoplasm and Polima cytoplasm were compared [14]. The numerous genome variations suggest different origins of the nap cytoplasm and Polima cytoplasm. Surprisingly, only four SNPs were noted when comparing the mitochondrial genomes of DH366 and Shaan 2A. This high congruency indicates the high quality of the mitochondrial genome obtained. For the far great sequencing depth of the plastid genome than the mitochondrial genome, we believe the plastid genome sequence have a high quality too.

## Conclusion

In this study, we developed a method to obtain plastid and mitochondrial genomes from plant tissue. This method comprises DNA extraction, DNA evaluation, DNA sequencing and de novo assembly. This method has many advantages, such as simple management, high efficiency, low cost, high accuracy and general applicability; however, the most important benefit is that plastid and mitochondrial genomes can be obtained simultaneously. Using this method, we obtained a high yield of high-quality cytoplasmic DNA, and the method provides reliable purity. The DNA can be used for sequencing on high-throughput sequencing platforms. Finally, the sequencing data can be assembled into complete plastid and mitochondrial genomes. Indeed, we obtained complete plastid and mitochondrial genomes from two additional rapeseed cultivars employing this method (unpublished data). For the simply procedure, we believe this method will promote research on plant cytoplasmic genome and related investigations.

## Supporting Information

File S1 **Details on the derivation of the VCQA equation.**
(DOC)
