# Supplementary material for: Effective Extraction and Assembly Methods for Simultaneously Obtaining Plastid and Mitochondrial Genomes
Source: PLoS One. 2014 Sep 24;9(9):e108291. doi: 10.1371/journal.pone.0108291 (PMC4177114; doi:10.1371/journal.pone.0108291)
Supplement: File S1 — Details on the derivation of the VCQA equation. (DOC) [file pone.0108291.s001.doc]

File S1 The details derivation of the VCQA equation

In the method GCNC, we use a control vector (pMD-ACR) containing three single copy genes (*β-actin*, which is used as reference gene; *ccmB* and *rpoB*, which are used as target genes) of rapeseed. These three genes possess the same copy number in the vector pMD-ACR. Therefore,

$\text{copy fold of target gene in control}=\text{copy fold of reference gene in control}$.

So, copy fold of target/reference in sample =$\frac{\text{copy fold of target gene in sample}}{\text{copy fold of reference gene in sample}}$

=$\frac{\text{copy fold of target gene in sample}\times\text{copy fold of reference gene in control}}{\text{copy fold of reference gene in sample}\times\text{copy fold of target gene in control}}$

=$\frac{\text{copy fold of target gene in sample}}{\text{copy fold of target gene in control}}\times\frac{\text{copy fold of reference gene in control}}{\text{copy fold of reference gene in sample}}$

=$\frac{\frac{\text{copy fold of target gene in sample}}{\text{copy fold of target gene in control}}}{\frac{\text{copy fold of reference gene in sample}}{\text{copy fold of reference gene in control}}}$.

For, copy fold of target gene=$\frac{\text{copy fold of target gene in sample}}{\text{copy fold of target gene in control}}$;

Copy fold of reference gene=$\frac{\text{copy fold of reference gene in sample}}{\text{copy fold of reference gene in control}}$.

So, copy fold of target/reference in sample =$\frac{\text{copy fold of target gene}}{\text{copy fold of reference gene}}$

=$\text{copy fold of target gene in sample/control}$.

As we know, the following equation has been wildly used in gene expression:

Copy fold of target gene in sample/control=,

Where E = efficiency of amplification, CT = number of cycles.

So, copy fold of target/reference in sample=.

In GCNC, the reference gene β-actin deputizes for copy number of nucleus DNA (ncDNA); the target genes ccmB and rpoB represent copy number of mitochondrial DNA (mtDNA) and chloroplast DNA (ptDNA) separately. So,

Copy fold of mtDNA/ncDNA in sample =;

Copy fold of ptDNA/ncDNA in sample =.
